# Effective and stable gene transduction in rhesus macaque iPSCs capable of T-lineage differentiation utilizing the piggyBac system

**DOI:** 10.1016/j.reth.2024.03.002

**Published:** 2024-03-18

**Authors:** Masahiro Tanaka, Yoshihiro Iwamoto, Bo Wang, Eri Imai, Munehiro Yoshida, Shoichi Iriguchi, Shin Kaneko

**Affiliations:** a Shin Kaneko Laboratory, Department of Cell Growth and Differentiation, Center for iPS Cell Research and Application (CiRA), Kyoto University, Kyoto, Japan; b Princess Margaret Cancer Centre, University Health Network, Toronto, Ontario, Canada

**Keywords:** iPSC, Rhesus macaque, piggyBac, T-cell differentiation

## Abstract

**Introduction:**

Genetically modified human induced pluripotent stem cell (iPSC)-based regenerative medicine has substantial potential in the treatment of refractory human diseases. Thus, preclinical studies on the safety and efficacy of these products are essential. Non-human primate (NHP) models such as the rhesus macaque are highly similar to humans in terms of size, lifespan, and immune system, rendering them superior models. However, effective gene transduction in rhesus macaque iPSCs (Rh-iPSCs) remains challenging. In this study, we investigated the effective gene transduction into Rh-iPSCs and its effect on differentiation efficiency.

**Methods:**

We established a gene transduction method using the piggyBac transposon vector system. Gene transduced Rh-iPSCs were analyzed for undifferentiated markers. We did teratoma assay to check pluripotency. Gene transduced Rh-iPSCs were differentiated into hematopoietic stem and progenitor cells (HSPCs) and T-cell lineage cells. Additionally, gene transduced Rh-iPSCs were compared the differentiation efficiency with parental Rh-iPSCs.

**Results:**

We could establish a gene transduction method using the piggyBac transposon vector system, demonstrating high efficiency and stable transgene expression in Rh-iPSCs. These Rh-iPSCs maintained long-term gene expression while expressing undifferentiated markers. Teratoma assay indicated that these Rh-iPSCs had pluripotency. These Rh-iPSCs could differentiate into HPSCs and T cells that express transgenes. These Rh-iPSCs can differentiate into hematopoietic stem cells and T cells that express transgenes. No significant differences in efficiency of differentiation were observed between parental Rh-iPSCs and these Rh-iPSCs.

**Conclusions:**

These results indicate that the piggyBac transposon vector is an excellent gene transfer tool for rhesus macaque iPSCs and could contribute to the advancement of preclinical studies using rhesus macaque iPSCs.

## Highlights

• PiggyBac transposon vector enables long-term gene expression in Rh-iPSCs.
• Gene-transduced Rh-iPSCs form teratomas.
• tEGFR-Rh-iPSCs maintain their expression after HSPC differentiation.
• tEGFR-Rh-iPSCs maintained tEGFR expression after differentiation into T cells.
• PiggyBac transposon vector as a simple method for gene transfection into Rh-iPSCs.

## 1 Introduction

Chimeric antigen receptor (CAR)-transduced T cells are effective against cancer [1]. Expanding T-cell therapy requires the development of “off-the-shelf” T-cell sources. Our previous study showed that CAR-transduced induced pluripotent stem cells (iPSCs) are expandable and can differentiate into functional T cells to suppress tumor cells in vitro and in vivo [[2], [3], [4], [5], [6]]. Furthermore, studies have demonstrated that the use of human leukocyte antigen (HLA)-edited iPSCs can effectively reduce both cost and time required for vein-to-vein manufacturing. These modified cells are capable of evading allogeneic immune rejection, and the inclusion of immune tolerance-inducing proteins such as CD47 and HLA-E can assist in developing hypoimmunogenic universal iPSCs [7,8].

To evaluate the safety and function of genetically modified iPSC-derived T cells, preclinical studies have used immunodeficient mouse models [6,9]. However, owing to significant differences in their characteristics compared to humans, accurate predictions cannot be achieved [10].

Non-human primate (NHP) models such as rhesus macaques are highly similar to humans in size, lifespan, and immune system, rendering them superior models for preclinical studies [11]. In the gene editing of rhesus macaque iPSCs, knockout of specific genes using the clustered regularly interspaced short palindromic repeats (CRISPR)/CRISPR-associated nuclease 9 (Cas9) system has been reported [12,13]. However, studies of efficient gene transfer methods are limited. Human immunodeficiency virus (HIV)-based lentiviral vectors are often used for gene transfer into human iPSCs [[4], [5], [6],^14^]. Although they can penetrate rhesus macaque cells, gene transduction cannot be established due to the presence of HIV resistance factors such as TRIM5a [15,16]. Moreover, there is a concern regarding the potential for unforeseen carcinogenesis in transgenic cells due to the ease with which genomic integrated viral vectors can be incorporated into proto-oncogenes or their promoter regions [17]. Also, retroviral transduction into primary rhesus macaque cells has been documented in various cell types such as T cells and hematopoietic stem and progenitor cells (HSPCs) [18,19]. This technique has been particularly employed in generating iPSCs from rhesus macaque fibroblasts [20]. However, it has been noted that in pluripotent stem cells, the silencing of retrovirus DNAs occurs rapidly [21,22].

The piggyBac transposon vector is a virus-independent gene delivery system that uses electroporation with a transposase vector. The advantages of the piggyBac transposon vector are that it can introduce large genes and because this system is virus-free, it is safe and easy for the operator to perform experiments [23]. Cell lines, including human iPS, have the ability to efficiently edit genes using the piggyBac transposon vector [24]. Additionally, the piggyBac transposon vector has been used to generate iPSCs from rhesus macaque fibroblasts [25].

We believe that the piggyBac transposon vector gene editing system could be a new gene transduction tool for rhesus macaque iPSCs (Rh-iPSCs).

In this study, we transduced marker genes into Rh-iPSCs using the piggyBac transposon vector/transposase vector system and generated Rh-iPSCs stably expressing these markers.

We selected two reporter proteins: emerald green fluorescent protein (EmGFP), which is frequently used for in vitro imaging [26], and truncated human epidermal growth factor receptor (tEGFR), which has low immunogenicity and is used as a transplanted cell marker [27].

## 2 Materials and methods

### 2.1 Cell culture

Rh-iPSC lines R1863 and R1887 were generated from T cells using a Sendai virus vector harboring KLF2, OCT3/4, SOX2, and c-MYC, as previously described [12]. Rh-iPSCs were maintained in Dulbecco's Modified Eagle's Medium/Nutrient Mixture F-12 Ham (Sigma-Aldrich, MO, USA) with 20% KnockOut serum replacement (Thermo Fisher Scientific, MA,USA), 1% l-Glutamine–penicillin–strepto-mycin solution (PSG, Sigma-Aldrich), MEM non-essential amino acids solution ( × 1) (Wako, Osaka, Japan), 5 ng/mL basic fibroblast growth factor (bFGF) (Wako), 10 mM 2-mercaptoethanol (Thermo Fisher Scientific), 3 μM CHIR99021 (TOCRIS bioscience, Bristol, UK), and 2 μM PD0325901 (Wako) with mouse embryonic fibroblasts (MEFs) as feeder cells.

### 2.2 PiggyBac transposon vector electroporation

The piggyBac transposon vector was purchased from VectorBuilder. pHL-EF1a-hcPBase-A was kindly provided by Dr. Hotta (CiRA, Kyoto, Japan). Rh-iPSCs were previously treated with 10 μM Y-27632 (Wako) for 1 h and then Rh-iPSCs were dissociated with Trypsin-EDTA solution (Sigma-Aldrich) into single cells. Single-cell suspension with 5 μg piggyBac transposon vector and 2 μg pHL-EF1a-hcPBase-A were electroporated by Maxcyte ExPERT ATx.

### 2.3 Flow cytometry and antibodies

The following antibodies were used in this study.

The anti CD3 (SP34-2), CD34 (563), NHP CD45 (D058-1283) antibodies were purchased from BD Biosciences (New Jersey, USA). The CD4 (OKT4), and EGER (AY13) antibodies were purchased from BioLegend (San Diego, CA). CD8β (SIDI8BEE) antibody was purchased from Invitrogen (Carlsbad, CA).

For cell staining, we used a BD FACSAria II or BD LSRFortessa (BD Biosciences). The data were analyzed using FlowJo software (Tree Star, Ashland, OR, USA). Single cells were analyzed by doublet discrimination, and dead cells were depleted by propidium iodide (Sigma-Aldrich) staining.

### 2.4 Immunofluorescence staining

Rh-iPSCs were fixed with 4% paraformaldehyde phosphate buffer solution (Wako). The cells were then stained with an anti-TRA-1-60 antibody (clone TRA-1-60, Millipore, MA, USA) or anti-SSEA-4 antibody (clone 813-70, Santa Cruz Biotechnology, TX, USA). The secondary antibody used was goat anti-mouse IgG (H + L) highly cross-adsorbed secondary antibody, Alexa Fluor™ 488 (Invitrogen). For nuclear staining, we used VECTASHIELD antifade mounting medium with DAPI (Vector Laboratories, CA, USA). Images were captured with fluorescence microscopy BZ-X810 (KEYENCE, Osaka, Japan).

### 2.5 Reverse transcription PCR (RT-PCR)

We generated complementary DNA from Rh-iPSCs using a High-Capacity cDNA Reverse Transcription Kit (Thermo Fisher Scientific). TaKaRa Ex Taq (TaKaRa Bio, Shiga, Japan) was used for RT-PCR. The bands were detected using a WSE-5400-CyP Printgraph Classic (ATTO, Tokyo, Japan).

### 2.6 Quantitative real-time PCR (qPCR)

Genomic DNA was extracted from Rh-iPSCs using NucleoSpin Tissue (TaKaRa Bio) according to the manufacturer's protocol. For the qPCR assay, we referred to previous report [28]. qPCR was performed using the StepOnePlus Real-Time PCR System (Thermo Fisher Scientific). Hypoxanthine phosphoribosyltransferase 1 (HPRT1) was used as a reference gene and quantified using THUNDERBIRD Next SYBR qPCR Mix (TOYOBO, Osaka, Japan). We used these primers: HPRT1 Forward Primer: 5′-TTATGGACAGGACTGAACGTCTTG-3′ and HPRT1 Reverse Primer: 5′-GCACACAGAGGGCTACAATGTG-3′.

To measure the 5’ inverted terminal repeat (ITR) region, we used THUNDERBIRD Probe qPCR Mix (TOYOBO). To make a standard curve, we used a serial dilution of plasmid (10^8^, 10^7^, 10^6^, 10^5^, 10^4^, 10^3^, 10^2^ and 10^1^ copies/μL).

Vector copy number (VCN) was calculated by following formula: VCN per cell = qPCR copy number/μL of vector target/qPCR copy number/μL of reference target × 2.

### 2.7 Teratoma formation

2 × 10^6^ Rh-iPSCs were suspended with 100 μL Corning Matrigel Basement Membrane Matrix (Corning, NY, USA) and 100 μL of cold D-PBS(−) (Nacalai Tesque, Kyoto, Japan). Rh-iPSCs were injected subcutaneously into the back just above the hind limb of 6-week-old female NOD. Cg-Prkdc^scid^ Il2rg^tm1Wjl^/SzJ (NSG) mice. The teratomas were dissected 8–10 weeks later, fixed with 10% formalin (Wako), and processed for hematoxylin and eosin staining. Images were captured with fluorescence microscopy BZ-X810 (KEYENCE).

### 2.8 In vitro T-Cell lineage differentiation from Rh-iPSCs via HSPCs

To obtain HSPCs, Rh-iPSCs were differentiated as previously reported [12]. In brief, small clumps of Rh-iPSCs were co-cultured on C3H10T1/2 feeder cells with Sac medium (Iscove's Modified Dulbecco's Medium (Sigma-Aldrich) consisting 15% fetal bovine serum (FBS), 1% PSG, insulin–transferrin–selenium (ITS-G) (1X) (Gibco), 450 mM monothioglycerol (Nacalai Tesque), 50 μg/mL ascorbic acid (Nacalai Tesque), in addition to 20 ng/mL vascular endothelial growth factor (VEGF) (R&D Systems, MN, USA) for 7 days. After 7 days, the cells were cultured in Sac medium supplemented with 30 ng/mL stem cell factor (SCF) (R&D Systems) and 10 ng/mL recombinant human Flt3-ligand (rhFlt3-L PeproTech, NJ, USA). After 14 days of culture, we obtained HSPCs from Sac-like structures derived from iPSCs.

Next, we transferred HSPCs onto OP9-DL1 cells and co-cultured in OP9 medium (aMEM consisting 15% FBS, 1% PSG, ITS-G (1X), 50 μg/mL ascorbic acid (Nacalai Tesque)) supplemented with 1 ng/mL recombinant human IL-7 (PeproTech) and 10 ng/mL rhFlt3-L. After 21 days culturing, we obtained CD8β, CD4 double positive T cells (DP cells).

### 2.9 Statistics

All statistical analyses were performed using the Prism software (GraphPad Software, CA, USA). In Fig. 5c and e, a two-sided Student's *t*-test was used to compare the two groups in parametric data. P > 0.05 was considered not significant.

## 3 Results

### 3.1 Gene transduction utilizing piggyBac transposon vector enables long-term gene expression in Rh-iPSCs while maintaining undifferentiated markers

**Fig. 1:**
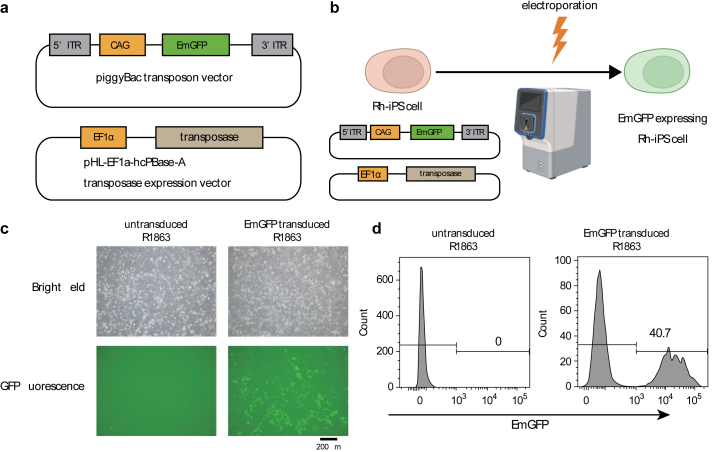
Flow chart for gene transduction into Rh-iPSCs and expression of EmGFP. a Schematic constructs of the piggyBac transposon vector and transposase-expressing vector. ITR: inverted terminal repeat, CAG: promoter. b Scheme of electroporation using the piggyBac system. Rh-iPSCs were dissociated into single cells and transduced with the piggyBac transposon and transposase-expressing vectors using a MaxCyte ATx electroporation device. Three days after electroporation, the cells were observed by fluorescence, and 7 days after electroporation, they were analyzed by flow cytometry. c Rhesus macaque iPSC line R1863 was transduced with a piggyBac transposon vector expressing EmGFP and a transposase-expressing vector by electroporation. After 3 days, EmGFP expression was observed using fluorescence microscopy. The scale bar indicates 200 μm. d Flow cytometry analysis of EmGFP expression 7 days after electroporation. The EmGFP-positive cells were sorted and cultured for several weeks.

First, the piggyBac transposon vector carrying EmGFP as a fluorescent marker and the piggyBac transposase-expressing vector pHL-EF1a-hcPBase-A were transduced into Rh-iPSCs (Fig. 1a). Three days after gene transduction, the expression of EmGFP was analyzed by fluorescence microscopy and flow cytometry, and approximately 40% of the iPSCs expressed GFP (Fig. 1b, c, d).

**Fig. 2:**
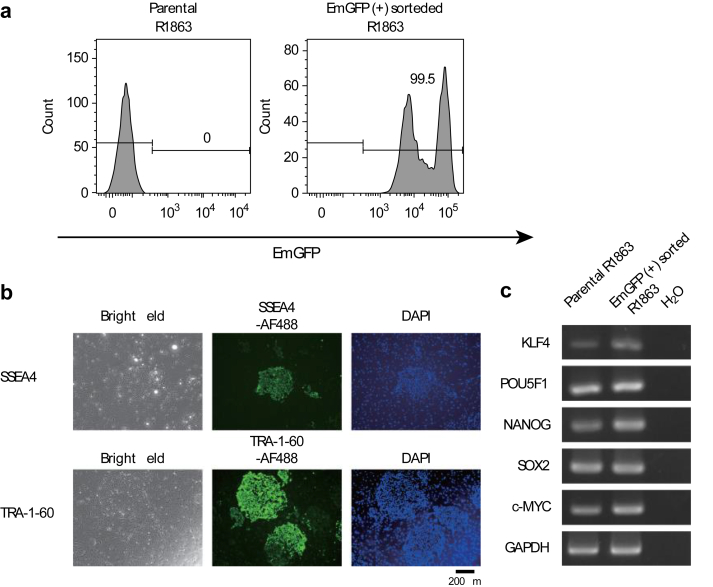
Character of EmGFP-transduced Rh-iPSCs by the piggyBac system. a EmGFP expression in sorted Rh-iPSCs The sorted Rh-iPSCs maintained EmGFP expression for 4 months at least. b Expression of undifferentiated markers SSEA4 and TRA-1-60 by immunofluorescence staining. The scale bar indicates 200 μm. c Comparison of pluripotency gene expression by RT-PCR between parental Rh-iPSCs and sorted EmGFP-transduced Rh-iPSCs

To purify transduced Rh-iPSCs, GFP-positive cells were sorted and cultured. Compared to the original iPSCs, almost all transgenic iPSCs were GFP-positive and they stably expressed GFP, even after 4 weeks (Fig. 2a). Gene transfer with the piggyBac transposon vector had no noticeable effect on the morphology of the transduced iPSCs or the undifferentiated cell surface markers SSEA4 and TRA-1-60 (Fig. 2b). Transcription factors Nanog, KLF4, POU5F1, SOX2, and c-Myc are mainly expressed in undifferentiated iPSCs. We examined the expression of these genes in transgenic and parental iPS using reverse transcription PCR (RT-PCR). Gene-transduced Rh-iPSCs and parental Rh-iPSCs maintained transcriptional expression.

**Fig. 3:**
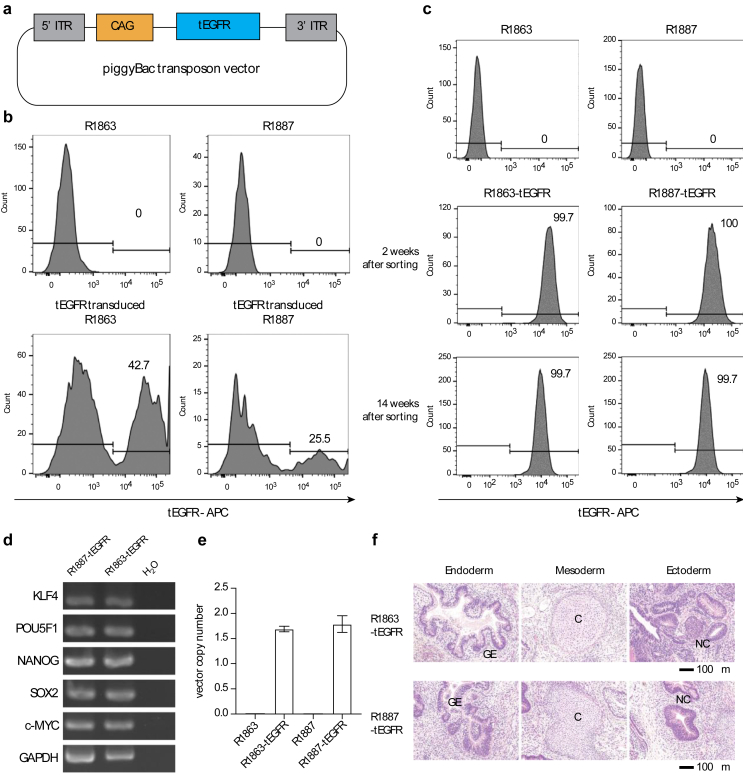
tEGFR gene transduction into Rh-iPSCs by the piggyBac system. a Schematic constructs of the piggyBac transposon vector expressing tEGFR. b Successful gene transduction in Rh-iPSC lines. tEGFR expression was analyzed using flow cytometry. tEGFR high expressing populations were sorted and cultured. c tEGFR expression in tEGFR-Rh-iPSCs The tEGFR-Rh-iPSC lines, R1863-tEGFR and R1887-tEGFR, maintained exogenous gene expression for 14 weeks at least. d Analysis of pluripotency genes by RT-PCR. e Transgene copy number of integration by the piggyBac system. f Teratoma formation assay. tEGFR-Rh-iPSCs differentiated into all three germ layers. GE, gut-like epithelium; C, cartilage; NC, neural crest. Scale bars indicate 100 μm.

Next, we generated a piggyBac transposon vector expressing tEGFR and attempted to transduce it into iPSCs (Fig. 3a). tEGFR was efficiently transfected into two Rh-iPSC clones (Fig. 3b). Sorting of cells with high tEGFR expression in each clone showed that tEGFR was still stably expressed after 14 weeks (Fig. 3c). The expression of Nanog, KLF4, POU5F1, SOX2, and c-Myc was confirmed by RT-PCR and was maintained after gene transfer (Fig. 3d). We named these Rh-iPSCs as tEGFR-Rh-iPS cells.

**Fig. 4:**
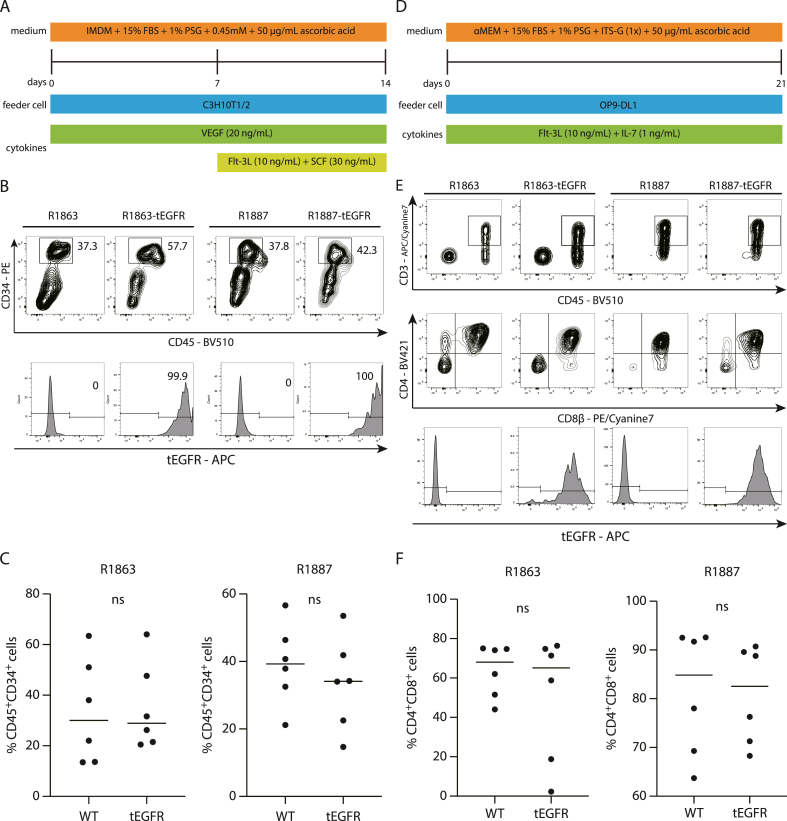
The differentiation into T-cell lineage cells from tEGFR-Rh-iPSCs. a Schematic illustration of differentiation from Rh-iPSCs to HSPCs. b Flow cytometric analysis of differentiated HSPCs based on CD34 and CD45 expression. HSPCs from tEGFR-Rh-iPSCs maintained tEGFR expression. c Comparison of the surface antigen profiles of HPCs. Each dot represents one biological replicate (n = 6). Non-paired Student's *t*-test. ns: not significant. d Schematic illustration of differentiation from HSPCs to T-cell lineage cells. e Flow cytometric analysis of differentiated T-cell lineage cells. f Comparison of the surface antigen profiles of DP cells. Each dot represents one biological replicate (n = 6). Non-paired Student's *t*-test. ns: not significant.

The copy number of tEGFR was measured by qPCR for each clone, and approximately one copy insertion was confirmed (Fig. 3e).

Taken together, Rh-iPSCs transduced with the piggyBac transposon vector maintained long-term and stable expression of transgenes while maintaining the expression of cell surface undifferentiated markers and transcription factors.

### 3.2 Gene-transduced Rh-iPSCs form teratomas

Untransfected Rh-iPSCs have been shown to form teratomas in vivo and to have pluripotency [12]. To test the pluripotency of tEGFR-Rh-iPSCs in vivo, we subcutaneously injected them into immunodeficient mice and observed their ability to form teratomas. The injected cells formed teratomas after 8 weeks. Histological examination revealed endodermal, mesodermal, and ectodermal tissues (Fig. 3f).

### 3.3 tEGFR-Rh-iPSCs maintain their expression after HSPCs differentiation

During iPSC differentiation, exogenous genes may be silenced through epigenetic changes. To test whether tEGFR-Rh-iPSCs could maintain tEGFR expression after differentiation into hematopoietic cells, we differentiated them into HSPCs with the support of 10T1/2 feeder cells (Fig. 4a). tEGFR-Rh-iPSCs differentiated into CD34-positive cells and parental cells, and maintained tEGFR expression (Fig. 4b, c).

### 3.4 tEGFR-Rh-iPSCs maintained tEGFR expression after differentiation into T cells

HSPCs derived from tEGFR-Rh-iPSCs were differentiated into CD8/CD4 double positive thymocytes (DP cells) (Fig. 4d). Both tEGFR-Rh-iPSCs and parental Rh-iPSCs differentiated into CD8b+/CD4+/CD3+/CD45+ cells and DP cells from tEGFR-Rh-iPSCs maintained tEGFR expression (Fig. 4e, f).

These results suggest that tEGFR gene transfer with the piggyBac system does not affect the T-lineage differentiation of iPSCs.

## 4 Discussion

In this study, we established a gene transduction method using a piggyBac system, demonstrating high efficiency and stable transgene expression in Rh-iPSCs. These iPSCs can differentiate into hematopoietic stem cells and T cells that express transgenes.

The piggyBac system is easy to manufacture as it is virus-free and requires less time than viral transduction. There is a concern regarding the potential for unforeseen carcinogenesis in transgenic cells due to the ease with which viral vectors are incorporated into proto-oncogenes or their promoter regions [29]. Analysis of gene insertion sites using the piggyBac system showed that the frequency of insertion around transcription start sites and CpG islands was significantly lower than that of retroviral vectors [29]. Additionally, the frequency of insertion into the genomic safe harbor is considerably higher than that of lentiviruses [29].

In the clinical application of iPSC-based medicines, it is necessary to predict their safety because unexpected side effects can occur and they are sometimes lethal to patients. This outcome is attributed to the preclinical study model conducted on mice considering the substantial differences in immune system and body size between humans and mice [30].

Rhesus macaques serve as valuable primate model animals in preclinical studies in humans owing to their anatomical and physiological similarities to humans [10,11,31]. Rhesus macaque iPSCs have been successfully generated from fibroblasts [20] and T cells [12] and differentiated into immune cells [12,32].

For clinical application of human iPSC-derived cells, it is necessary to produce homogeneously differentiated cells from genetically engineered iPSCs. Genome editing using the CRISPR-Cas9 system and gene transfer using lentiviral vectors are being practiced [5,14]. In previous studies on Rh-iPSCs, genome editing was performed using the CRISPR-Cas9 system [12,13]; however, gene transduction by lentiviral vectors has not been reported. We attempted gene transduction into Rh-iPSCs with a HIV-based lentiviral vector but were unable to confirm gene transfer. This could be attributed to the resistance of rhesus macaque cells toward HIV infection [15,16]. There are some improved HIV-based lentiviral vectors and Simian immunodeficiency virus (SIV)-based lentiviral vectors, but they are not easy to access [16,33]. Moreover, lentiviral vector transduction of HSPCs in rhesus macaques has been suggested to induce aberrant clonal hematopoiesis [34].

Therefore, we used the piggyBac transposon vector as a simple method for gene transfection into Rh-iPSCs. Compared to lentiviruses, the piggyBac transposon vector can introduce a large gene (>10 kbp), and because a plasmid is used, the method is simple and does not require viral containment.

Rh-iPSCs transduced with piggyBac transposon vectors maintained high gene expression even after several months of culture and silencing did not occur (Fig. 2). Considering that piggyBac transposon vectors are used for reprogramming Rh-iPSCs, this suggests that the piggyBac transposon vector is an outstanding tool for gene introduction into rhesus macaque cells [24,25].

The teratomas assay indicated that the grafted tEGFR-Rh-iPSCs differentiated into three germ layers and showed multilineage differentiation. We did not examine tEGFR expression in any of the three germ layers. Nevertheless, validating its expression would broaden the applicability of this transduction system across various tissue types.

Gene-engineered adoptive cell therapy, typified by CAR-transduced T-cell therapy, has shown great promise as a new cancer therapy. However, there are several issues, including the difficulty in obtaining a sufficient quantity of cells for transplantation, the time-intensive manufacturing process, and the associated high costs. iPSCs have an unlimited self-renewal capacity and iPSC-derived cytotoxic T cells proliferate efficiently upon repeated stimulation. Therefore, iPSCs are expected to serve as a source of cells for T-cell therapy.

We differentiated tEGFR-Rh-iPSCs into HSPCs and then into T-cell lineages. tEGFR-Rh-iPSC-derived differentiated cells maintained tEGFR expression.

For clinical applications, iPSCs must be cultured in the absence of feeder cells and cultured and differentiated under defined component conditions. In this study, we used mouse embryonic fibroblasts (MEF) and 10T1/2 and OP9 cells originating from mouse cells. A recent report showed Rh-iPSC feeder-free generation and cultivation under chemically defined conditions [25], and our group reported that human iPSCs can differentiate into killer T cells under feeder-free and chemically defined conditions [6]. By advancing these technologies, we expect to establish an Rh-iPSC differentiation method.

In our future studies, we plan to generate target gene-expressing T cells derived from genetically modified Rh-iPSCs. The expected genes were CAR and endogenous T-cell receptor (TCR). Functional assays can be performed in vitro and in vivo by inducing the expression of these proteins. A previous report has shown that anti-CD20 CAR-transduced peripheral T cells cause serious adverse effects [35]. In a phase I trial of piggyBac-modified CD19 CAR-T therapy conducted in Australia piggyBac-modified CD19 CAR-T cells from transplant donors were administered to 10 patients with relapsed B-cell tumors post-hematopoietic stem cell transplantation. Although the therapy demonstrated high efficacy, with reported remission in all 10 treated patients, two patients developed CAR-T cell-derived T-cell lymphoma [36]. It was reported that the CAR-T cell-derived T-cell lymphoma cells had a relatively high CAR gene copy number (24 copies). While no specific integration into cancer gene regions was observed in the analysis of CAR gene insertion sites, integration into the BACH2 gene region, considered a tumor suppressor gene, was observed in both cases. The BACH2 gene is a transcription factor associated with cutaneous T-cell lymphoma, and its expression is suppressed in CD4-positive T cells. Since the mechanism are still unclear, it was concluded that follow-up is important [37].

For our piggyBac-based iPSC transduction system, we are planning to conduct careful assessments, including checking the copy number and specific integration into cancer gene regions, as well as mutations in genes related to T-cell lymphoma. These analyses will help us select safe iPSC clones for further investigation. Moreover, because of the easiness of gene editing, iPS cells can carry suicide genes such as tEGFR [38]. Therefore, in the event of a serious adverse reaction, it can be expected to be eliminated immediately.

SIV is a model HIV that infects rhesus macaques. Research on antigen-specific TCR against SIV-expressing T cells is progressing, and effective TCRs have been cloned [39]. Therefore, we generated Rh-iPSCs that express TCR against SIV and differentiate into killer T cells. The generated T cells will be transplanted into SIV-infected rhesus macaques, and their therapeutic effect and safety will be evaluated.

## 5 Conclusion

The piggyBac system is a highly effective gene transfer tool for rhesus macaque iPSCs. These results are expected to substantially contribute to the advancement of preclinical studies on rhesus macaque iPSCs.

## Credit authorship contribution statement

Masahiro Tanaka: Conceptualization, Data curation, Formal analysis, Investigation, Methodology, Project administration, Resources, Validation, Visualization, Roles/Writing - original draft, Writing - review & editing. Yoshihiro Iwamoto: Conceptualization, Data curation, Formal analysis, Investigation, Methodology, Project administration, Resources, Visualization. Bo Wang: Investigation, Supervision, Writing - review & editing. Eri Imai: Investigation, Validation. Munehiro Yoshida: Investigation. Shoichi Iriguchi: Conceptualization, Methodology, Supervision, Writing - review & editing. Shin Kaneko: Funding acquisition, Project administration, Resources, Supervision, Writing - review & editing.

## Declaration of competing interest

The authors declare the following financial interests/personal relationships which may be considered as potential competing interests: Shin Kaneko is a founder, shareholder, and chief scientific officer at Thyas, Co., Ltd., and received research funding from Takeda Pharmaceutical, Co., Ltd., Kirin Holdings, Co., Ltd., Astellas, Co., Ltd, Terumo Co., Ltd., Tosoh, Co., Ltd., and Thyas, Co., Ltd.
